# Infrared thermographic changes after decompression surgery in patients with carpal tunnel syndrome

**DOI:** 10.1186/s12891-023-06193-4

**Published:** 2023-01-31

**Authors:** Yeo Eun Park, Sang-Eok Lee, Yoon Sik Eom, Jae Man Cho, Joong Won Yang, Man Su Kim, Heum Dai Kwon, Jang Woo Lee, Dougho Park

**Affiliations:** 1 Department of Physical Medicine and Rehabilitation, National Health Insurance Service Ilsan Hospital, 10444 Goyang, Republic of Korea; 2 Department of Rehabilitation Medicine, Pohang Stroke and Spine Hospital, 37659 Pohang, Republic of Korea; 3 Department of Orthopedic Surgery, Pohang Stroke and Spine Hospital, 37659 Pohang, Republic of Korea; 4 Department of Neurosurgery, Pohang Stroke and Spine Hospital, 37659 Pohang, Republic of Korea; 5 Department of Medical Science and Engineering, School of Convergence Science and Technology, Pohang University of Science and Technology, Pohang, Republic of Korea

**Keywords:** Carpal tunnel release, Carpal tunnel syndrome, Temperature, Thermography, Vasomotor activity

## Abstract

**Background:**

Digital infrared thermal imaging (DITI), which detects infrared rays emitted from body surface to create a body heat map, has been utilized at various musculocutaneous conditions. Notably, DITI can demonstrate autonomic vasomotor activity in the nerve-innervated area, and thus may be of use in carpal tunnel syndrome (CTS). In this study, we compared DITI findings before and after carpal tunnel release (CTR) surgery in patients with unilateral CTS to investigate the corresponding neurophysiological changes.

**Methods:**

In this retrospective cohort study, DITI parameters such as the temperature differences between the median and ulnar nerve territories and median nerve-innervated digital anisometry were measured. Subjective symptom duration, pain scale, and ultrasonographic findings were also compared before and after CTR. Patients were evaluated before and 6 weeks after CTR, respectively.

**Results:**

A total of 27 patients aged 59.0 ± 11.2 years were finally included. After CTR, median nerve-innervated thermal anisometry was improved (2.55 ± 0.96 °C to 1.64 ± 1.34 °C; *p* = 0.003). The temperature differences between the median and ulnar nerve territories were not significantly changed. Subjective pain, the Simovic Weinberg Clinical Scale, and palmar bowing of the flexor retinaculum were also significantly improved (*p* <  0.001 for all comparisons).

**Conclusions:**

Our results demonstrated that DITI findings could reflect an improvement in autonomic function after CTR. Therefore, DITI can be an objective method to assess pre- and post-operative neurophysiologic changes in CTS.

## Background

Carpal tunnel syndrome (CTS) is one of the most common peripheral nerve entrapment syndromes which is caused by compression of the median nerve within the carpal tunnel [1]. Elevated carpal tunnel pressure and traction of median nerve can cause nerve injury, obstruction of venous outflow, and ischemia [2]. A recent anatomic study also suggests that myofascial stress affecting paraneural sheath plays a role in pathogenesis of CTS [3]. Also carpal tunnel syndrome is associated with repeated wrist use, pregnancy, diabetes, and systemic diseases such as hypothyroidism [4]. Symptoms include sensory changes such as tingling, abnormal sensation, and burning of the wrist and fingers along the innervation of the median nerve, and motor symptoms such as thenar atrophy and weakness. It is more common in women than in men and is known to occur bilaterally in more than 50% of cases [5].

Most CTS patients with mild to modest symptoms receive conservative treatments. However, severe CTS patients or those with nerve lesions proved by electrodiagnosis often require surgery [6]. Carpal tunnel release (CTR) is generally considered in cases which fail to respond to conservative treatment, develop weakness and atrophy of the thenar muscles, and traumatic CTS [7]. Previous studies have reported that more than 20% of patients with CTS underwent CTR [8, 9].

Although there is no definitive standard for the diagnosis of CTS, electrodiagnostic testing is widely used in conjunction with clinical symptoms. In addition to physical examinations such as Tinel’s sign and Phalen’s maneuver, methods such as US, magnetic resonance imaging, and infrared body thermography are used [10–12]. Infrared body thermography (DITI) detects infrared rays emitted from the surface of the human body and displays it as a body heat map according to the temperature differences. Specifically, DITI is known to be able to evaluate the function of sympathetic vasomotor fibers regulating cutaneous blood flow, which cannot be adequately tested by electrodiagnostic tests [13] . As a relatively inexpensive and non-invasive test, it is currently used for some pain-related diseases such as entrapment neuropathy, spinal neuropathy, and complex regional pain syndrome [14].

Several studies using DITI have been published in previous studies on CTS; unfortunately, they did not show consistent results, [15–17] including pre- and postoperative cases [18–20]. In our previous study, we reported that DITI findings may differ depending on the severity and symptom duration of CTS. As DITI reflects the state of the autonomic nervous system, the possibility of using DITI in CTS is plausible [21].

In this context, we aimed to identify the thermographic, clinical, and sonographic changes before and after CTR in patients with unilateral CTS. Subsequently, we primarily investigated the significance of DITI in the evaluation and understanding of neurophysiological changes after CTR.

## Methods

### Patients and assessments

This retrospective cohort study was performed from January 2020 to July 2021 and used a database from a single hospital’s electrodiagnostic laboratory. Symptoms, signs and laboratory findings were compared before and after surgery in patients who had undergone surgical treatment for unilateral CTS. All patients showed clinical and electrodiagnostic findings consistent with CTS, and significant symptoms that were difficult to improve with conservative treatment, such as pharmacological or injection treatment. Patients with diseases that may affect DITI; including central nervous system lesions involving the affected hand, lower cervical radiculopathy, systemic peripheral neuropathy, peripheral vascular disease, arthritis of the hand, previous history of hand or wrist surgery; and systemic diseases such as tumor, thyroid diseases, fibromyalgia, and uncontrolled diabetes; were excluded. The study was approved by the local institutional review board (Approval number: PSSH0475–202201-HR-002-01).

The degree of pain was recorded through the numerical rating scale (NRS) before surgery, and Simovic Weinberg Clinical Scale [22] was used to rate the severity of symptoms. Electrodiagnostic testing was performed; based on nerve conduction studies and needle EMG findings, disease severity was classified under Steven’s classification [23] as mild, moderate, or severe.

Ultrasonography was performed using a linear probe (12–5 MHz) in the proximal carpal tunnel between the pisiform and scaphoid bones. Patients were examined in a sitting position, with the elbow flexed at 90 degrees and the forearm fully supinated. The cross-sectional area and palmar bowing of the median nerve were measured at the site, respectively.

### Digital infrared thermographic parameters

The DITI test was performed according to the same protocol as previously published by these authors, and the same parameters were evaluated [21]. The IRIS-8000® (Medicore, Seoul, Korea) was used to record the thermographic image. Before electrodiagnosis, DITI was performed under the following conditions: the room temperature was maintained at 23–25 °C; the subject was dressed in loose clothing and allowed to acclimate to the environment for 15–20 min; the subject was placed 1.5 m from the thermographic camera; no lotion or ointment was applied before the test; topical patches, splints, and metal accessories were removed; vigorous exercise and physical therapy were to be avoided within 4 h of the test; no alcohol or caffeine consumption, or smoking were allowed within 12 h of the test.

We designated six regions of interest of the thermal image of the palmar surface. Skin temperature was measured at the center of the finger pulp of the first, second, third, and fifth digits based on an area of 400 data points. Additionally, measurements were obtained from the thenar and hypothenar eminences based on an area of 800 data points. The mean value of all data points in the study area was determined. By translating the approved red-green-blue data to temperature, the final result was displayed in degrees Celsius. We created the indicators below for data interpretation and quantitative analysis. The values were calculated by substituting the average body temperature obtained within each region of interest.

- Temperature difference between the median and ulnar nerve digits (ΔM−U digits)
  - = (digit 1 + digit 2 + digit 3)/3−digit 5
- Temperature difference between the thenar and hypothenar areas (Δthenar−hypothenar)
  - = thenar − hypothenar,
- Temperature difference between the median and ulnar nerve territories (ΔM−U territories)
  - = ΔM−U digits + Δthenar–hypothenar,
- Median nerve-innervated digits anisometry
  - = │digit 1−digit 2│ + │digit 1−digit 3│ + │digit 2−digit 3│

### Surgical procedures

All patients underwent open CTR. The surgeon first made a 2 cm incision in the central part of the distal wrist. After dissecting the subcutaneous layer, the transverse carpal ligament was identified. Thereafter, decompression was performed by incision of the transverse carpal ligament while checking the median nerve sheath. Then, the surgeon removed adhesion tissues around the median nerve and confirmed that the nerve was sufficiently decompressed. Finally, each layer was sutured closed. Brachial plexus blockade was done prior to surgery.

Except for electromyography, NRS, SCS, ultrasonography, and DITI were evaluated according to the same protocol by the same evaluator 6 weeks after surgery.

### Statistical analysis

Paired t-test was performed for each parameter before and after surgery. All statistical analyses were conducted using SPSS 22.0 (IBM, Armonk, NY, USA).

## Results

### Baseline characteristics

A total of 27 patients were included in this study; 7 men and 20 women with an average age of 59 ± 11.2 years. There were more participants with right hand than left hand involvement (right hand involvement: 59.3%). The average duration of symptoms was 12 ± 13.85 months. According to the electrodiagnostic severity grading, 3 patients were classified as moderate and 24 as severe; no patients were considered as mild (Table 1).

**Table 1:** Demographic characteristics of participants

	Total (***N*** = 27)
Age, years	59.0 ± 11.20
Male, N (%)	7 (25.9)
Involved hand, right, N (%)	16 (59.3)
Body mass index, kg/m2	26.5 ± 3.5
Duration of symptom, months	12.0 ± 13.6
Electrodiagnostic classification	
Moderate, N (%)	3 (11.1)
Severe, N (%)	24 (88.9)

### Clinical and sonographic findings

As a result of the study, the pain NRS score in the patients decreased from 6.07 ± 1.21 points before surgery to 2.07 ± 1.03 points 6 weeks after surgery (*p* <  0.001); and the Simovic Weinberg Clinical Scale changed from 25.44 ± 2.75 points before surgery to 6.67 ± 3.66 points (*p* <  0.001). Ultrasound findings of the median nerve cross-sectional area (CSA) in the proximal carpal tunnel was not significantly different from 0.20 ± 0.04 cm^2^ to 0.18 ± 0.08 cm^2^ before and after surgery, respectively (*p* = 0.191). However, a significant reduction in PB was observed, changing from 4.10 ± 0.71 mm to 3.24 ± 0.91 mm before and after surgery, respectively (*p* <  0.001) (Table 2).

**Table 2:** Clinical and ultrasound parameters before and 6 weeks after carpal tunnel release

Parameter	Before CTR^a^	6 weeks after CTR	***p***-value
NRS^b^ of pain	6.07 ± 1.21	2.07 ± 1.03	< 0.001
SWCS^c^	25.44 ± 2.75	6.67 ± 3.66	< 0.001
US^d^ findings			
CSA^e^ of median nerve, cm^2^	0.20 ± 0.04	0.18 ± 0.08	0.191
Palmar bowing, mm	4.10 ± 0.71	3.24 ± 0.91	< 0.001

^a^ CTR, carpal tunnel release; ^b^NRS, numerical rating scale of pain; ^c^SWCS, Simovic and Weinberg Clinical Scale; ^d^US, ultrasound; ^e^CSA, cross-sectional area of the median nerve

### Digital infrared thermographic findings

There was no statistically significant difference in both the skin temperature of each finger, except for the fourth finger (mediated by both median and ulnar nerves), and that of the thenar and hypothenar areas before and after surgery. Other processed parameters, including ΔM − U digits, Δthenar−hypothenar, and ΔM − U territories, showed no statistically significant differences before and after surgery. Only median nerve-innervated digits anisometry showed a significant decrease after surgery compared to before surgery (2.55 ± 0.96 °C to 1.64 ± 1.34 °C; *p* = 0.003) (Table 3).

**Table 3:** Values of infrared thermography before and 6 weeks after carpal tunnel release

Parameter	Before CTR^a^	6 weeks after CTR	***p***-value
Skin temperature of measured area			
1st digit, °C	28.80 ± 1.38	28.30 ± 1.62	0.082
2nd digit, °C	28.14 ± 1.16	27.83 ± 1.54	0.250
3rd digit, °C	28.06 ± 1.41	27.73 ± 1.76	0.214
5th digit, °C	27.65 ± 1.74	27.26 ± 2.04	0.171
Thenar, °C	29.89 ± 1.09	30.40 ± 2.18	0.228
Hypothenar, °C	29.88 ± 1.26	29.84 ± 1.21	0.821
Processed parameter			
ΔM − U digits^b^, °C	0.68 ± 0.95	0.69 ± 0.95	0.946
Δthenar−hypothenar^c^, °C	0.01 ± 0.82	0.57 ± 1.67	0.125
ΔM − U territories^d^, °C	0.69 ± 1.48	1.26 ± 2.12	0.157
Anisometry^e^, °C	2.55 ± 0.96	1.64 ± 1.34	0.003^*^
│digit 1 − digit 2│	0.93 ± 0.49	0.60 ± 0.54	0.011^*^
│digit 1 − digit 3│	1.12 ± 0.52	0.73 ± 0.73	0.028^*^
│digit 2 − digit 3│	0.50 ± 0.34	0.32 ± 0.33	0.012^*^

Table showing mean and standard deviation

^a^CTR, carpal tunnel release; ^b^ ΔM-U digits, temperature difference between the median and ulnar nerve digits; ^c^ Δthenar-hypothenar, temperature difference between the thenar and hypothenar areas; ^d^ ΔM-U territories, temperature difference between the median and ulnar nerve territories; ^e^ Anisometry, anisometry of the median nerve-innervated digits

* *P* < 0.05

## Discussion

In this study, we demonstrated the decreased median nerve-innervated digital thermal ansiometry after CTR. This finding was in accordance with the postoperative improvement of clinical symptoms and US findings. In CTS, the increased interstitial fluid pressure in the carpal tunnel or the pressure that directly compresses the median nerve changes the microvasculature of the median nerve, resulting in ischemia and blood flow disorders [24]. Damage to unmyelinated small fibers in the early stages of CTS may increase thermal variation in areas innervated by the same nerve through mechanisms such as sympathetic dysfunction, reactive vasoconstriction, and antidromic sensory fiber activation [25]. Furthermore, a neuroanatomic study recently identified superficial fascia to be innervated by autonomic and sensory nerve fibers, which suggests the role of superficial fascia stress in CTS [26]. We have reported in a previous study that as the severity of CTS worsens, the anisometry also gradually increases [21]. Anisometry of median-nerve innervated digits might suggest that as disease progresses, damage in thin-unmyelinated fibers cause disregulaton of vasomotor activity, thereby resulting in thermal variations within the same nerve-innervated area.

Infrared thermographic changes were observed, along with pain reduction and clinical improvement after CTR in this study. It can be inferred that the statistically significant decrease in anisometry values after CTR in this study is due to the improvement of blood flow and autonomic function in the median nerve-innervated area after decompression of the carpal tunnel. On the other hand, there was no significant change in skin temperature other than anisometry after surgery. Specifically, secondary parameters, such as ΔM − U digits, Δthenar−hypothenar, and ΔM − U territories did not show significant changes after surgery. According to our previous study, these parameters, as opposed to anisometry, stand out in the early stage of the disease, but become less apparent as the severity and duration of the disease increases [21]. Those who underwent surgery were electrodiagnostically severe in most cases (88.9%), with an average prolonged symptom duration of 12.0 months. Therefore, the indicators which showed a marked difference in the early stage of the disease did not change much postoperatively. An exception to this was in the case of anisometry, which became more prominent as the disease progressed, demonstrating improvement after the operation. There have been previous studies which have identified autonomic changes before and after CTR by utilizing the advantages of DITI. Ming et al. [18] observed changes in DITI findings before, and 6 months after, CTR in 22 patients with CTS. Similar to our findings, they reported a decrease in median nerve-innervated digits anisometry. However, their results showed that median-ulnar territory difference significantly diminished, which is discordant to our study. Ming et al. do not mention disease severity, unlike our study where the patients may be a more severe and homogenous group. ΔM − U territories values from Ming et al. in CTS patients tended to be higher than that of healthy controls or than that of the participants in our study, and ΔM − U territories is a parameter that diminishes as the disease progresses [21]. Another study by Baic et al. [19] compared the temperature differences of the thenar area and eight sites of median nerve-innervated digits. While their study analysis on digits was too segmented, they also demonstrated better temperature distribution within the median territory after CTR compared to the thenar area. Meanwhile, Bargiel et al. [20] performed three DITI tests on 40 patients who underwent CTR 2 days before and 2 weeks after surgery. As a result, they reported alleviation of clinical symptoms after carpal tunnel surgery, but no improvement in thermographic findings. However, Bargiel et al. used a different methodology than our study, and only examined overall hand temperature before and after cooling by dynamic thermography. Subsequently, previous studies between CTR and DITI findings have shown inconsistent results; further studies to identify neurophysiologic changes after CTR are still lacking. This study’s results can be validated because the study parameters are based on our previous study, which reported on the possibility of using DITI in CTS; in particular, we primarily suggested that DITI reflects the state of the autonomic nervous system and shows different findings depending on the severity and symptom duration of CTS [21].

Since the median nerve contains most of the sympathetic nerves of the hand [27], it is thought that autonomic dysfunction may occur in CTS [28]. Therefore, there have been studies using various approaches to evaluate autonomic dysfunction caused by median nerve compression in CTS. Methods such as sympathetic skin response (SSR), vasomotor response by Doppler technique, and capillaroscopy are used to evaluate sympathetic activity [29, 30]. Among them, capillaroscopy and Doppler have rarely been performed in CTS. While several studies on sympathetic skin response in CTS patients have been conducted, their results were inconsistent. Although there was no difference between affected and unaffected hands in some studies [30], others have shown decreased sympathetic activity in CTS [31–33]. In a postoperative study, the SSR before and after CTR failed to reflect post-surgical improvement in one study [34], yet in another study, the SSR amplitude ratio was restored after surgery [35]. The use of SSR in CTS is limited as it has not been established which of several parameters such as amplitude, median/ulnar latency difference, SSR waveform, etc., has diagnostic value in CTS. Therefore, DITI is considered the only objective tool to be able to assess the function of the small nerve fibers of CTS.

Electrodiagnostic testing is widely used in clinical practice as a tool to confirm CTS. However, while neuro-electrophysiological examination shows a strong correlation with the functional aspect of the median nerve, there are cases where there is no clear correlation with the symptoms of actual patients [36]. Moreover, there is also a study which found that there is no significant correlation with the degree of improvement of clinical symptoms after surgery [34]. In the early stages of nerve injury, damage to the unmyelinated small fibers, which conduct electrical impulses of the autonomic nervous system, occurs first. However, electrical diagnostic testing is limited with regards to reflecting the damage to these small fibers [37]. In addition, ultrasonography is also a widely used modality; it can directly check the morphology of the damaged nerve with the naked eye, examine both sides simultaneously, enable dynamic study, and can be used to guide treatment administration via injection [38]. Regarding the postoperative US findings of CTS, El-Karabaty et al. [39] reported that the flattened median nerve recovered to its normal shape after carpal tunnel decompression. Naranjo et al. [40] reported that the prognosis of surgery is good when the difference in the median nerve cross-sectional area before and after surgery is small. However, US can only confirm anatomical abnormalities and does not directly reflect the functional state or level of nerve damage. In our study, the degree of PB decreased after CTR, however, this is a direct anatomical change due to CTR. The CSA, which objectively determines the extent of median nerve swelling, did not show any significant changes after surgery. Therefore, it did not accurately reflect the improved pain level and clinical symptoms after surgery.

This study has several limitations. This study is a retrospective study with single-center’s database and further validation studies are needed. Another objective test, an electrodiagnostic test, could not be performed after surgery, hence analysis thereof was not done. It unconfirmed whether there were other symptoms related to vasomotor function. Since DITI abnormalities are interpreted as being related to thin unmyelinated nerve fiber dysfunction, it is necessary to investigate whether there are other subjective complaints related to this in future studies.

## Conclusions

Carpal tunnel decompression surgery improved anisometry between median nerve-innervated digits in digital infrared thermal imaging, which is thought to be due to the improvement of vasomotor activity and blood flow mediated by the autonomic nervous system. This study suggests that digital infrared thermal imaging could possibility be of use as an objective evaluation method after carpal tunnel release.

## Data Availability

The datasets used and/or analyzed during the current study are available from the corresponding author on reasonable request.

## Abbreviations

CTR: Carpal tunnel syndrome
CTS: Carpal tunnel surgery
DITI: Digital infrared thermal imaging
NRS: Numerical rating scale of pain
SWCS: Simovic and Weinberg Clinical Scale
US: Ultrasound
CSA: Cross-sectional area
